# Chronicity and Mental Health Service Utilization for Anxiety, Mood, and Substance Use Disorders among Black Men in the United States; Ethnicity and Nativity Differences

**DOI:** 10.3390/healthcare6020053

**Published:** 2018-05-23

**Authors:** Vickie M. Mays, Audrey L. Jones, Susan D. Cochran, Robert Joseph Taylor, Jane Rafferty, James S. Jackson

**Affiliations:** 1 Departments of Psychology and Health Policy and Management, University of California, Los Angeles Fielding School of Public Health, Los Angeles, CA 90095, USA; 2 UCLA Center for Bridging Research Innovation, Training and Education for Minority Health Disparities Solutions, Los Angeles, CA 90095, USA; cochran@ucla.edu; 3 Informatics, Decision-Enhancement and Analytic Sciences Center (IDEAS 2.0), Veteran Affairs Salt Lake City Health Care System, Salt Lake City, UT 84148, USA; Audrey.Jones@hsc.utah.edu; 4 Department of Internal Medicine, University of Utah School of Medicine, Salt Lake City, UT 84132, USA; 5 Departments of Epidemiology and Statistics, University of California, Los Angele Fielding School of Public Health, Los Angeles, CA 90095, USA; 6 School of Social Work, University of Michigan, Ann Arbor, MI 48109, USA; rjtaylor@umich.edu; 7 Program for Research on Black Americans, Institute of Social Research, Ann Arbor, MI 48106, USA; jrafferty@umich.edu (J.R.); jamessj@umich.edu (J.S.J.); 8 Department of Psychology, University of Michigan, Ann Arbor, MI 48109, USA; jamessj@umich.edu

**Keywords:** psychiatric disorders, anxiety, mood, depression, substance use disorders, blacks, gender, ethnicity, nativity, Bipolar I and II, Dysthmia

## Abstract

This study investigated ethnic and nativity differences in the chronicity and treatment of psychiatric disorders of African American and Caribbean Black men in the U.S. Data were analyzed from the National Survey of American Life, a population-based study which included 1859 self-identified Black men (1222 African American, 176 Caribbean Black men born within the U.S., and 461 Caribbean Black men born outside the U.S.). Lifetime and twelve-month prevalence of DSM-IV mood, anxiety, and substance use disorders (including Bipolar I and Dysthmia), disorder chronicity, and rate of mental health services use among those meeting criteria for a lifetime psychiatric disorder were examined. Logistic regression models were employed to determine ethnic differences in chronicity, and treatment utilization for disorders. While rates of DSM-IV disorders were generally low in this community sample of Black men, their disorders were chronic and remained untreated. Caribbean Black men born in the U.S. had higher prevalence of Post-Traumatic Stress Disorder, Major Depressive Disorder, and Alcohol Abuse Disorder compared with African American men. Foreign born Caribbean Black men experienced greater chronicity in Social Phobia and Generalized Anxiety Disorder compared to other Black Men. Utilization of mental health service was low for all groups of Black Men, but lowest for the foreign born Caribbean Black men. Results underscore the large unmet needs of both African American and Caribbean Black men in the United States. Results also highlight the role of ethnicity and nativity in mental disorder chronicity and mental health service utilization patterns of Black men.

## 1. Background

Historically, population-based studies of mental health disorders in Non-Latino Blacks have found that the rates of disorders are lower than or equal to those of Non-Latino Whites [1,2,3]. We have learned in the last couple of decades predominantly from the National Survey of American Life that these disorders may vary considerably by nativity and ethnic subpopulation membership [4]. While in general rates of mental health disorders are low in Blacks as a group particularly for Black men, Caribbean Black men have been found to show an elevated burden of mood, anxiety and substance use disorders compared to African American men [5,6,7].

Despite this finding of lower or equal rate of disorders when Blacks are diagnosed with mental health disorders, they are also more likely than Whites to rate their disorders as more severe, disabling and persistent in nature [1,6,7]. Due to the low rates of help-seeking and use of mental health treatment by Black men in the United States, our knowledge [8] about the chronicity of their disorders is nascent and in need of additional study.

Population-based studies of mental health needs of Black men have examined rates of psychiatric disorders among Black fathers [9], correlates of depression among African American men [10,11,12], and barriers to professional help-seeking in this population [13,14]. Moreover, we are increasingly gaining an appreciation of ethnic and nativity variations in mental health needs in the U.S.

The Patient Protection and Affordable Care Act (ACA) presented an opportunity to improve the treatment of psychiatric disorders for racial and ethnic minorities. Expansions of health insurance and investments in the primary care and safety net systems are likely to provide a new pathway to treatment for African American and Caribbean Black men [15,16,17,18]. With these opportunities, improved understandings of ethnic and nativity variations in mental health service needs are required to inform the development of health services interventions that will ultimately reduce racial/ethnic disparities and improve the health and well-being of Black men in the U.S regardless of whether it is the ACA or some other approach to integrated mental health services.

## 2. Study Aim

The current study aimed to investigate ethnic and nativity differences in the chronicity and treatment of psychiatric disorders among Black men in the U.S. including Bipolar I and II. We used data from the National Survey of American Life (NSAL) to provide nationally-representative estimates of the prevalence and chronicity of psychiatric disorders, as well as mental health services use for African American and U.S. and foreign born Caribbean Black men with DSM-IV mood, anxiety, and substance use disorders. Epidemiological information regarding the prevalence and chronicity of psychiatric disorders across immigrant and non-immigrant groups of Black men is important for identifying cultural and environmental factors that contribute to Black men’s mental health. Moreover, such information is needed to develop targeted interventions to reduce the burden of untreated mental disorders in African American and Caribbean Black men.

## 3. Methods

### 3.1. Study Design

We used data from the National Survey of American Life (NSAL) a cross sectional survey of DSM-IV psychiatric disorders and mental health service utilization among Black Americans [19]. The sampling frame for the survey used a four-stage probability sampling design [20]. The core sample is nationally representative of individuals living in households located in the contiguous United States where at least one Black adult (age 18 or older) resides. To recruit persons of Caribbean descent, geographic areas with a high density (at least 10%) of Caribbean Blacks was also included in the sampling frame. Participants were interviewed between January 2001 and March 2003 with response rates of 70.7% for African Americans and 77.7% for Caribbean Blacks. Population-based weights were created to account for the unequal probability of selection, nonresponse, and post-stratification [21]. 

### 3.2. Analytical Sample

The NSAL includes 6072 individuals including 1271 African American men and 643 Black men of Caribbean descent. For the current study, we excluded 33 African American men who were born outside of the U.S. because the sample size was too small for analyses. We also excluded 16 African American men and 6 Caribbean Black men because their nativity status was missing. The analytical sample in this study included 1222 African American men born within the U.S., 176 Caribbean Black men born within the U.S., and 461 Caribbean Black men born outside of the U.S.

### 3.3. Measures

#### 3.3.1. Psychiatric Disorders

The NSAL assessed the presence of probable lifetime and 12-month psychiatric disorders using the World Mental Health version of the World Health Organization’s Composite International Diagnostic Interview (WHO-CIDI) [22,23]. The WHO-CIDI is a fully structured interview used to assess the prevalence of DSM-IV psychiatric disorders. In the current study, we examined patterns of five anxiety disorders (General Anxiety Disorder, Panic Disorder, Agoraphobia, Social Phobia, Post-Traumatic Stress Disorder; PTSD), four mood disorders (Major Depressive Disorder, Dysthymia, Bipolar Disorder I and II), and four substance use disorders (Alcohol Abuse, Alcohol Dependence, Drug Abuse, and Drug Dependence). In addition, we investigated lifetime and twelve-month prevalence of a panic attack.

#### 3.3.2. Race and Ethnicity

Participants were grouped by race and ethnicity based on the racial/ethnic classification in the U.S. Census. For the current study, we use the term, Black, to refer to those participants who by self-report labeled themselves as Black. We also use Caribbean Black to refer to participants who both identified as Black and reported ancestral ties to a Caribbean country. African American refers to participants who identified as Black, were born in the U.S., and who did not report ancestral ties to the Caribbean.

#### 3.3.3. Chronicity

Disorder chronicity was defined as the proportion of adults with a lifetime psychiatric disorder that met criteria in the twelve months prior to the interview [6,24]. The sample was restricted to the 486 Black men who met criteria for a lifetime psychiatric disorder and who reported an age of onset at least two years prior to the interviews. This measure is a proxy for identifying those with a disorder lasting more than twelve months [6,7].

We coded participants as positive for lifetime mental health service utilization if they indicated ever seeking help for nerves, emotions or mental health, or for use of alcohol or drugs from a medical or mental health provider. In this study we included formal health service providers including general medical providers (general practitioners, medical specialist (e.g., cardiologist), medical professionals (e.g., nurse), and specialty mental health providers (e.g., psychologist, psychiatrist, counselor, or social worker in a mental health setting).

### 3.4. Statistical Analysis

The analyses were conducted in four stages. First, we used cross-tabulations to investigate prevalence estimates of lifetime and prior twelve-month disorders, chronicity, and mental health services use across the three groups of men. We present weighted study proportions and standard errors adjusted for the complex survey design.

Second, we used multivariate logistic regression models to compare the prevalence of psychiatric disorders (lifetime and twelve-month disorders) among men varying in U.S. nativity and Caribbean heritage. In these analyses, we compared the adjusted odds of meeting the DSM-IV disorder criteria among Caribbean groups compared to African American men. We then evaluated nativity differences in adjusted odds of meeting the DSM-IV criteria among Caribbean Black men (the African Americans were excluded). The lifetime and twelve-month prevalence analyses controlled for participant characteristics known to vary in risk for a psychiatric disorder [3]: age, household income, poverty status (a ratio of family income to the U.S. census poverty threshold in 2001), education, employment status, marital status, and geographic region of the U.S.

Third, we used logistic regression analyses to examine group differences in the chronicity of each measured disorder. The sample was restricted to those participants whose disorder began at least two years prior to the interview. In these analyses we compared the odds of meeting the twelve-month criteria for a disorder among the Caribbean groups compared to U.S. born African American men. We then evaluated nativity differences among Caribbean Black men (the African Americans were excluded). These analyses controlled for sociodemographic characteristics identified as being associated with chronicity [25,26,27,28,29]: age (18–30, 31–50, 51+), education (high school or less vs. other), marital status (married vs. other), and poverty status (household income below 100% federal poverty level vs. other). Additionally, the analyses controlled for the age of disorder onset [6].

Lastly, we used logistic regression models to examine group differences in mental health services use among those who met lifetime and twelve-month criteria for each anxiety, mood, and substance use disorders. These models controlled for predisposing, enabling, and need factors which are commonly associated with access and use of mental health services [30,31,32]: age (18–30, 31–50, 51+), education (high school or less vs. other), marital status (married vs. other), poverty status (household income below 100% federal poverty level vs. other), and insurance status at the time of the interview.

All analyses were conducted using SAS, a statistical program that uses the Taylor expansion approximation technique to estimate variances given the complex sampling design of the NSAL [33]. The survey weights were employed to account for the unequal probability of selection and nonresponse, and for post-stratification. Because the Caribbean Black sample is smaller and more clustered than the African American sample, the standard errors for Caribbean Black men are often larger than those for African American men.

## 4. Results

There were notable differences in the distribution of sociodemographic characteristics of African American and U.S. and foreign born Caribbean Black men (see Table 1). Caribbean Black men born in the U.S. tended to be younger on average than African American men. Further, both U.S. and foreign born Caribbean Black men, as compared to African American men, tended to live in households with higher levels of income. Rates of employment were highest among foreign born Caribbean Black men and lowest among the African American men. Foreign born Caribbean Black men were also most likely to be married or cohabiting at the time of the NSAL interview. In contrast, U.S. born Caribbean Black men reported the highest rates of being single or never married and African American men reported the highest rates of being previously married (separated, divorced, or widowed). Compared to African Americans, both groups of Caribbean Black men tended to report higher levels of education (some college or greater). Finally, consistent with residential patterns in the United States [34], a greater proportion of African American men resided in the South when compared to both groups of Caribbean Black men. The majority of participants were covered by health insurance at the time of the interview.

### 4.1. Lifetime DSM IV Psychiatric Disorders

Approximately 30% of Black men overall met criteria for at least one of the measured lifetime psychiatric disorders (see Table 2). This differed among the three groups of men. Specifically, U.S. born Caribbean Black men had significantly higher rates of meeting criteria for any of the measured lifetime disorders compared to both the African American and the foreign born Caribbean Black men. In addition, U.S. born Caribbean Black men were also most likely to meet criteria for two or more lifetime disorders.

For the most part, this pattern of findings was repeated when anxiety, mood, and substance use disorders were considered separately. Overall, 14% of men met criteria for a lifetime anxiety disorder, 10% for a lifetime mood disorder, and 18% for a lifetime substance use disorder. Rates were significantly higher for any anxiety disorder and any substance use disorder among U.S. born Caribbean Black men as compared to either the U.S. born African Americans or foreign born Caribbean Black men. U.S. born Caribbean Black men had significantly higher rates of Major Depressive Disorder and significantly lower rates of Bipolar Disorder compared to African American men. The rates of Post-Traumatic Stress Disorder and Panic Disorder appeared particularly high among the U.S. born Caribbean Black men, though these ethnic differences were not statically significant after adjusting for sociodemographic characteristics.

Rates of specific psychiatric disorders for the foreign born Caribbean Black men were generally comparable to those of African Americans with the exception of substance use disorders. Foreign born Caribbean Black men showed the lowest rate of substance use disorders with just 7% meeting the criteria for any lifetime substance use disorder compared with one in three U.S. born Caribbean Black men and one in five U.S. born African American men.

### 4.2. Twelve-Month DSM IV Psychiatric Disorders

Approximately one out of eight Black men (12%) met twelve-month criteria for a psychiatric disorder. U.S. born Caribbean Black men were the most likely to meet criteria for the presence of at least one recent disorder, and this group was also significantly more likely than foreign born Caribbean Black men to meet criteria for two or more recent disorders.

For the sample as a whole, prevalence of recent psychiatric disorders was 7% for any anxiety disorder, 5% for any mood disorder, and 4% for any substance use disorder. U.S. born Caribbean Black men were significantly more likely than both of the other two groups to meet criteria for any twelve-month anxiety or mood disorder. Significant differences were specifically observed in Post-Traumatic Stress Disorder (PTSD), Panic Disorder, Panic Attack, and Major Depressive Disorder.

Within the Caribbean samples, U.S. born men had higher rates of twelve-month Dysthymia and any substance use disorder compared to foreign born Caribbean Black men. We were unable to obtain adjusted group differences in the rates of alcohol abuse, alcohol dependence, drug abuse, and drug dependence disorders between the U.S. and foreign born Caribbean Black men due to the sparse rates of substance use in these groups. None of the foreign born Caribbean Black men in the current study met criteria for alcohol abuse or drug dependence in the twelve months prior to the interview, and the rates of alcohol dependence and drug abuse in this group were less than one percent.

### 4.3. Chronicity of DSM IV Psychiatric Disorders

Four of ten Black men with a lifetime psychiatric disorder continued to meet DSM-IV criteria in the twelve months prior to the interview. Among the entire sample, 49% of those with a lifetime anxiety disorder, 51% of those with a lifetime mood disorder, and 22% of those with a lifetime substance use disorder continued to meet criteria in the twelve months prior to the interview.

Caribbean Black men born in the U.S. more frequently evidenced chronicity among the measured disorders compared to African American men. However, the pattern of ethnic and nativity differences in disorder chronicity varied for specific disorders (see Table 3). U.S. born Caribbean Black men evidenced chronic Panic Disorder more frequently than foreign born Caribbean Black men. In contrast, the foreign born Caribbean Black men more frequently evidenced chronic Social Phobia compared to both U.S. born groups. African American men tended to have lower rates of chronic anxiety disorders and higher rates of chronic substance use disorders compared to foreign born Caribbean Black men. The bivariate analyses indicated ethnic differences in the chronicity of Agoraphobia or Bipolar Disorder (*p* < 0.01). However, small samples precluded the use of multivariate models to identify adjusted group differences in chronicity for these disorders. Finally, we were unable to estimate ethnic variations in the chronicity of specific substance use disorders since none of the foreign born Caribbean Black men with lifetime alcohol abuse or lifetime drug dependence disorder continued to meet criteria for these conditions in the twelve months prior to the interview.

### 4.4. Lifetime Mental Health Services Utilization

Approximately half (52%) of all Black men who met the criteria for any psychiatric disorder during their lifetime also reported ever speaking with a medical or mental health provider about mental health concerns (see Table 4). Rates of treatment contact for the full sample were 59%, 59%, and 52% among those with any mood disorder, any anxiety disorder, or any substance use disorder, respectively.

The rates of service use among Black men with any lifetime psychiatric disorder were 65% for African American men, 76% for Caribbean Black men born in the U.S., and 81% among foreign born Caribbean Black men. While these rates appear comparable, there were significant ethnic variations in mental health services use among Black men with specific psychiatric disorders. Of those who met the DSM-IV criteria for Post-Traumatic Stress Disorder or Generalized Anxiety Disorder during their lifetime, U.S. born Caribbean Black men were more likely than the U.S. born African American men to report speaking with a provider about mental health concerns.

For those men with a positive lifetime history of a Panic Disorder, U.S. born African American men had significantly higher rates of service use compared to foreign born Caribbean Black men. Lastly, among men who met criteria for a substance use disorder during their lifetime, foreign born Caribbean Black men were more likely than U.S. born Caribbean Black men to report speaking to a provider regarding mental health or substance use issues.

### 4.5. Twelve-Month Mental Health Service Use

Finally, we investigated rates of mental health services use of Black men who met criteria for a mood, anxiety, or substance use disorder in the twelve months prior to the interview. Just one quarter (27%) of Black men with a recent disorder had sought mental health care from a medical or mental health provider in the past year. There were significant differences in twelve-month services use. Seven percent of foreign born Caribbean Black men with a disorder reported services use in the past year compared to 48% of U.S. born Caribbean Black men and 27% of U.S. born African Americans, *p* < 0.05.

## 5. Discussion

The current study sought to determine ethnicity and nativity differences in mental health experiences of African American and Caribbean Black men in the U.S. To achieve this goal, we provided nationally representative estimates of the prevalence and chronicity of DSM-IV mood, anxiety, and substance use disorders, and mental health services use among African American and U.S. and foreign born Caribbean Black men. The mental health needs of Black men have not always been visible as this population is less likely than others to seek treatment in traditional primary care and specialty mental health settings [8,13,35].

Our study found that, although the prevalence of most DSM-IV disorders is low in community-dwelling samples of Black men, disorders in this population are often chronic and untreated. Moreover, there were significant ethnic and nativity variations in the prevalence, chronicity, and treatment of *DSM-IV* mood, anxiety, and substance use disorders which may provide clues towards the etiology and persistence of disorders in Black men.

### 5.1. Extra Risk of U.S. Born Caribbean Black Men

U.S. Born Caribbean Black men experienced elevated prevalence of PTSD, Panic Disorder, MDD, and Alcohol and Drug Use Disorders compared to African American Men. While prior studies have reported that approximately one in 12 Caribbean Black men are likely to meet the criteria for PTSD [36], our study found that the lifetime prevalence of this disorder is almost twice as high with 1 in 7 Caribbean Black men born in the U.S. meeting criteria for PTSD. Our findings correspond with others which have found elevated rates of DSM-IV disorders [9,37], suicide attempts [38] and psychiatric hospitalization [39] among Caribbean Black men born in the U.S. For example, Doyle and colleagues found that Caribbean fathers in the U.S. had elevated rates of psychiatric disorders compared to African American fathers [9].

This study expands upon prior investigations of Black men’s mental health by providing nationally representative estimates of chronicity, and mental health service use as well as the prevalence of Bipolar Disorder I and Dysthymia for Black men. Although rare, the lifetime and twelve-month prevalence of Bipolar Disorder I was higher among African American men compared with Caribbean Black men. Because there is a history of misdiagnosis of affective disorders and over-diagnosis of schizophrenia in African Americans [40,41] particularly men, it is important to provide clinicians with the training and resources needed to accurately identify affective disorders including Bipolar I disorders in African American populations.

### 5.2. Most Disorders Are Chronic

This study extends prior investigations of the persistence of psychiatric disorders among Non-Latino Blacks [6,7] and provides new findings of ethnic and nativity variations in the chronicity of specific psychiatric disorders among African American and Caribbean Black men. Results of our study finds Caribbean Black men born in the U.S. more frequently evidenced chronic Generalized Anxiety Disorder (GAD), Panic Disorder, and Substance Use Disorders than the other groups of Black men. Interestingly, U.S. born Caribbean Black men with a lifetime history of GAD and Alcohol Abuse were more likely than African American men with the same disorders to report lifetime mental health services use. 

Results from other literature into Black men’s mental health may help to explain the patterns of ethnic and nativity differences in chronicity observed here. In an early study that was among the first to notice persistence (i.e., chronicity) of mental disorders in Black men, it was thought that this chronicity is potentiated by factors that are concurrent with or subsequent to the onset of disorders [1]. Some have suggested that the experience of race-based discrimination could be a contributing factor in mental health outcomes [42,43,44]. In a study we conducted of the relationship between perceived discrimination and mental health services in the primary care setting we found that, for Black Americans, when they perceived discrimination within the context of these services they were more likely to drop out of treatment prematurely [45]. While there has been much research documenting that race-based discrimination is associated with mental health consequences, we may need to look beyond just the association with the disorders themselves and starting look at how these experiences of discrimination may serve to complicate the recovery process. In the Mays et al. study, Blacks were more likely to drop out of treatment than Whites, which clearly would serve to support persistence of their mental health problems. In a study by Mereish et al. [46], the association of discrimination with depressive symptoms was mediated by the self-esteem in African American men, but not in Caribbean men. The authors postulate that differences between African American and Caribbean men in their responses to discriminatory experiences may be a function of racial socialization as well as the appraisal of what constitutes race-based discrimination. While the current study did not assess race-based discrimination or racial socialization, these may be important areas of inquiry in future studies focused on elucidating ethnic and nativity differences in the chronicity of disorders among Black men. 

Our findings raise important questions about the need to explore factors that may drive U.S. born Caribbean Black men to seek mental health services more readily than other Black men. Given the potentially elevated psychiatric morbidity experienced by U.S. born Caribbean Black men, future studies are needed to elucidate what contributes to the persistence of psychiatric disorders in this population as it delays the recovery and resiliency process.

### 5.3. Mental Health Services Use Was Subpar for All Black Men

Only half of Black men who met the criteria for a psychiatric disorder in this study had ever spoken with a healthcare provider about mental health or substance use concerns. The rates were not much higher (62%) among those with two or more comorbid disorders. Prior studies have indicated that the low rates of mental health services use among Black men may be attributed to economic barriers, discrimination in the healthcare setting, provider mistrust, and low perceptions of need [35,45,47,48,49]. It is possible that the low rates of mental health services use observed in the current study are due, in part, to discontinuous insurance coverage or lack of a usual source of care [50,51,52]. As noted above, when Black men use general medical services, they are more likely than Whites to perceive experiences of discrimination in the healthcare encounter. Thus, mental health service use may also be as a function of how Black men perceive systems of care to work for them, as well as the extent to which their racial socialization plays a role in what they perceive to be the likelihood and consequences of discrimination in receiving mental health services [45].

While masculinity norms could prevent some men from utilizing mental health services [13], literature into Black men’s help-seeking found that Black men will reach out for help with emotional problems; they are likely to use informal systems of care [48,49]. Thus, some of the Black men with a psychiatric disorder in the current study are likely to have sought mental health assistance from clergy, family or friends, or other informal sources of support [14,53,54,55,56,57,58,59,60]. Given the chronic and persisting nature of psychiatric disorders, which has been reported for Blacks more than others [6,7], our findings indicate that Black men’s mental health needs are not being met adequately through these alternate pathways to care. Gender and culturally specific interventions may be required to improve access, use and effectiveness of mental health treatment for African American and Caribbean Black men.

### 5.4. Limitations

The study findings should be interpreted with four limitations in mind. First, the sample sizes were small when rates of treatment were broken down by ethnicity, nativity status, and specific disorders. This restricted our power to only detect differences which may have been present with larger samples. Second, our estimates of the lifetime and twelve-month prevalence of alcohol and drug dependence disorders may be underestimated as the WHO-CIDI used diagnostic criteria that did not strictly adhere to those provided by the DSM-IV [61,62]. Third, the estimates here only generalize to non-institutionalized Black men. Individuals that are homeless, incarcerated, or living on a military base were excluded from the study. Fourth, we were unable to examine group differences in past year services use among those with specific twelve-month disorders due to the small number of men meeting criteria for a recent disorder and the low proportion of those men with services use. As an example, none of the 18 foreign born Caribbean Black men with a recent panic attack had spoken with a provider about mental health or substance use concerns in the twelve months prior to the interview. Similarly, none of the ten U.S. born Caribbean Black men with a recent drug use disorder had spoken with a provider in the twelve months prior to the interview.

### 5.5. Specific Considerations and Future Research

As much of our information about the mental health status, needs and service utilization of African American and Caribbean men comes from non-institutionalized populations it is important to remember that we have little to no data for the mental health needs of the estimated over 1 million Black men in jails and incarcerated settings [63,64]. African American men are more than six times as likely as White men to be incarcerated [65], and rates of psychiatric disorders are higher among incarcerated Black men compared to those in the community [66,67]. Moreover, the leading cause of death among Black men in jails is suicide [68]. These men are often released into communities who are unprepared to care for their mental health problems, under-resourced in general for mental health providers, and lacking information on the ways in which a history of incarcerations adds to their mental health recovery [63]. It is important to attend to these sampling considerations when interpreting the low prevalence rates of disorders found in non-institutionalized samples of African American men and when planning for population level service needs. A balanced research agenda that includes institutionalized Black men will address some of the myths of low prevalence of mental health disorders and underscore the high levels of unmet service needs for Black and Caribbean males.

Additional research is needed to isolate the underlying factors, such as acculturative stress or exposure to discrimination, that contribute to elevated rates of PTSD, panic disorder, and MDD among Caribbean Black men born in the U.S. There is also a need to study whether integrated care and its’ treatment approach result in increased or decreased likelihood of treatment and whether that treatment approach given the system of care in place contributed to reducing chronicity of mental disorders in Black men in the United States.

## 6. Conclusions

Our study provides new findings on the prevalence of Bipolar I and Dysthymia, the chronicity of psychiatric disorders, and patterns of mental services use for DSM-IV mood, anxiety, and substance use disorders among African American and U.S. and foreign born Caribbean Black men. The integrated care approach presents a unique opportunity to expand access to mental health services particularly for Black men in the United States [15]. In light of these opportunities, improved understandings of ethnic and nativity variations in mental health service needs are necessary [69,70]. Mental health screening efforts in the primary care setting must be gender and culturally specific to improve the early identification and treatment of disorders for African American and Caribbean Black men and to mitigate the chronic course of their disorders [71].

## Figures and Tables

**Table 1:** Demographic Characteristics of African American vs. Caribbean U.S. and Foreign Born Men in the National Survey of American Life, 2001–2003.

	African American	Caribbean Black		
Sociodemographic Characteristics	U.S. Born *n* = 1222	U.S. Born *n* = 176	Foreign Born *n* = 461	
	Weighted % (SE)			Rao-Scott X^2^
Age				
18–29	24.3 (1.7)	52.5 (3.8)	24.7 (3.8)	
30–44	36.2 (1.3)	26.5 (2.7)	32.3 (4.3)	
45–59	24.2 (1.4)	12.0 (3.2)	24.5 (6.5)	
60 or greater	15.4 (1.2)	9.0 (5.6)	18.4 (3.9)	21.68 **
Income				
Less than $18,000	23.6 (1.8)	19.6 (5.7)	16.8 (3.9)	
$18,000–$31,999	23.1 (1.2)	21.4 (5.7)	20.8 (2.9)	
$32,000–$54,999	28.2 (1.4)	15.2 (5.4)	26.3 (3.3)	
$55,000 or greater	25.1 (1.9)	43.9 (7.7)	36.1 (4.6)	16.40 *
Employment Status				
Working	70.5 (1.5)	76.0 (4.0)	80.7 (3.4)	
Not Working	29.5 (1.5)	24.0 (4.0)	19.3 (3.4)	8.34 *
Marital Status				
Currently Married	49.1 (1.6)	39.8 (7.7)	70.8 (4.2)	
Previously Married	20.4 (1.4)	8.9 (2.1)	11.2 (3.1)	
Never Married	30.5 (1.7)	51.2 (8.0)	18.1 (2.7)	36.83 ***
Education				
Less than High School	23.7 (1.6)	27.9 (10.6)	18.0 (3.3)	
High School	40.0 (1.7)	21.5 (4.8)	32.8 (3.6)	
Some College	22.9 (1.6)	22.9 (7.2)	23.5 (4.5)	
College	13.4 (1.4)	27.7 (6.6)	25.7 (4.0)	18.92 **
Poverty Status				
Household income below poverty level	17.1 (1.6)	12.2 (3.6)	13.3 (3.8)	
Household income above poverty level	82.9 (1.6)	87.8 (3.6)	86.7 (3.8)	0.78
Geographic Region				
South	57.3 (2.8)	17.0 (3.6)	38.8 (11.5)	
Non-South	42.7 (2.8)	83.0 (3.6)	61.2 (11.5)	14.86 ***
Insurance Status				
Insured	81.8 (1.0)	79.4 (4.2)	78.0 (2.4)	
Uninsured	18.2 (1.0)	20.6 (4.2)	22.0 (2.4)	2.21

* *p* < 0.05, ** *p* < 0.01, *** *p* < 0.001. SE—standard error.

**Table 2:** Weighted Prevalence of Lifetime and Twelve-Month Psychiatric Disorders among African American vs. U.S. and Foreign Born Caribbean Black Men in the National Survey of American Life, 2001–2003.

DSM-IV Disorder	Lifetime DSM-IV Disorders			Twelve-Month DSM-IV Disorder		
	African American	Caribbean Black		African American	Caribbean Black	
	U.S. Born *n* = 1222	U.S. Born *n* = 176	Foreign Born *n* = 461	U.S. Born *n* = 1222	U.S. Born *n* = 176	Foreign Born *n* = 461
	Weighted % (SE)			Weighted % (SE)		
**Anxiety Disorders**						
Agoraphobia	1.7 (0.5) _a_	-- _a_	2.1 (1.1) _b_	0.7 (0.3) _a_	-- _b_	0.3 (0.3) _a,b_
Post-traumatic Stress Disorder	5.1 (0.7)	15.9 (7.4)	4.3 (1.9)	2.4 (0.5) _a_	14.4 (6.4) _b_	3.0 (1.8) _a_
Social Phobia	6.8 (0.8)	8.8 (3.3)	5.7 (2.5)	3.3 (0.6)	5.2 (3.8)	5.0 (2.5)
General Anxiety Disorder	2.9 (0.7)	4.4 (3.2)	2.1 (0.8)	1.3 (0.4)	3.4 (3.1)	1.6 (0.8)
Panic Disorder	2.5 (0.6)	11.5 (7.4)	1.7 (0.6)	1.5 (0.4) _a_	9.7 (7.7) _b_	1.0 (0.5) _a,b_
Panic Attack	17.2 (1.6) _a_	37.2 (8.9) _b_	17.7 (4.8) _a_	5.4 (0.7) _a_	29.6 (10.3) _b_	6.4 (4.0) _a_
Any Anxiety Disorder	13.6 (1.3) _a_	30.5 (5.8) _b_	12.8 (3.9) _a_	6.7 (1.0) _a_	23.2 (5.9) _b_	9.0 (4.2) _a_
**Mood Disorders**						
Major Depressive Disorder	8.8 (0.9) _a_	21.1 (6.5) _b_	8.9 (2.8) _a,b_	4.6 (0.7) _a_	16.4 (5.6) _b_	4.9 (1.7) _a_
Dysthymia	2.7 (0.5)	8.0 (5.8)	1.7 (0.9)	1.9 (0.5) _a,b_	7.6 (5.9) _a_	0.9 (0.8) _b_
Bipolar Disorder I, II	2.3 (0.5) _a_	0.9 (0.5) _b_	1.0 (0.5) _a,b_	1.7 (0.5) _a_	0.2 (0.2) _b_	0.9 (0.5) _a,b_
Any Mood Disorder	9.9 (1.0)	21.4 (8.2)	9.3 (2.8)	5.1 (0.7) _a_	16.5 (8.2) _b_	5.3 (2.3) _a_
**Substance Disorders**						
Alcohol Abuse ^‡^	15.9 (1.1) _a_	32.6 (8.0) _b_	6.7 (2.3) _c_	3.7 (0.7)	13.9 (9.2)	--
Alcohol Dependence ^‡^	5.4 (0.7) _a,b_	10.5 (5.2) _a_	3.0 (2.6) _b_	1.9 (0.5)	5.1 (3.8)	0.1 (0.1)
Drug Abuse ^‡^	10.4 (1.1) _a,b_	19.7 (8.1) _a_	4.0 (2.5) _b_	1.6 (0.5)	8.5 (5.7)	0.2 (0.2)
Drug Dependence ^‡^	4.1 (0.8) _a,b_	6.9 (3.5) _a_	3.2 (2.6) _b_	1.3 (0.4)	0.8 (0.5)	--
Any Substance Disorder	18.4 (1.3) _a_	33.1 (7.9) _b_	7.3 (2.3) _c_	4.4 (0.7) _a_	15.0 (9.0) _a_	0.3 (0.2) _b_
**Any of the Above Disorders**	30.5 (1.5) _a_	52.5 (8.2) _b_	19.6 (4.1) _c_	12.2 (1.1) _a_	38.5 (9.2) _b_	11.1 (3.6) _a_
**2 or More of Above Disorders**	17.0 (1.2) _a_	35.9 (9.2) _b_	9.7 (3.5) _c_	5.8 (0.7) _a,b_	16.6 (8.3) _a_	4.3 (2.2) _b_

Subscripts (a, b, c) indicate pairwise comparison results using multivariate logistic regressions. Groups with different subscripts are significantly different from each other. For example, African American men have significantly lower rates of Major Depressive Disorder compared to U.S. born Caribbean Black men as they do not share a subscript. However, both groups share a subscript with the foreign born Caribbean Black men indicating that the rates are not significantly different at the *p* < 0.05 level. No subscript indicates that there are no significant differences in the rate of disorder across the three groups. -- unable to estimate prevalence estimate because there were zero cases that met the disorder criteria. ^‡^ Multivariate pairwise comparisons of ethnic differences in the specific twelve-month prevalence rates were not conducted due to the small samples of Caribbean Black men. SE—standard error.

**Table 3:** Weighted Percent of African American vs. Caribbean U.S. and Foreign Born Men with A Lifetime DSM-IV Disorder That Also Met Criteria in the Twelve Months Prior to the Interview.

		African American	Caribbean Black	
DSM-IV Disorder		U.S. Born	U.S. Born	Foreign Born
	N	Weighted % (SE)		
**Anxiety Disorders**				
Agoraphobia ^‡^	24	33.0 (5.3)	--	13.8 (0.4)
Post-traumatic Stress Disorder	63	47.6 (7.1)	91.0 (9.6)	83.5 (12.8)
Social Phobia	111	47.9 (6.4) _a_	59.6 (23.0) _a_	88.5 (7.0) _b_
General Anxiety Disorder	50	41.8 (9.8) _a_	78.3 (5.5) _a,b_	75.4 (11.3) _b_
Panic Disorder	45	54.6 (9.1) _a,b_	84.3 (17.4) _a_	56.1 (6.7) _b_
Any Anxiety Disorder	156	46.0 (3.8)	76.0 (9.7)	74.4 (13.1)
**Mood Disorders**				
Major Depressive Disorder	136	49.0 (5.9)	77.7 (14.1)	54.0 (16.7)
Dysthymia ^‡^	41	69.0 (8.9)	95.5 (6.1)	53.2 (19.2)
Bipolar Disorder I, II ^‡^	22	56.9 (9.4)	26.2 (0.0)	88.5 (0.4)
Any Mood Disorder	151	49.1 (5.7)	76.8 (14.2)	53.7 (16.7)
**Substance Use Disorders**				
Alcohol Abuse	237	20.9 (3.2) _a_	42.8 (20.8) _a_	-- _b_
Alcohol Dependence ^‡^	76	32.0 (5.2)	48.5 (39.4)	2.6 (2.5)
Drug Abuse	149	14.5 (3.7)	43.1 (27.7)	1.3 (1.6)
Drug Dependence ^‡^	60	29.9 (7.9)	8.9 (6.7)	--
Any Substance Disorder	270	21.3 (3.2) _a_	45.3 (25.0) _a,b_	1.8 (1.2) _b_
**Any of the Above Disorders**	475	36.6 (2.9) _a_	73.4 (7.2) _b_	55.8 (8.8) _a,b_

Analyses restricted to those that first met the DSM-IV criteria at least two years prior to the interview. ^‡^ Multivariate pairwise comparisons of ethnic differences in the chronicity specific disorders were not conducted due to the small samples. -- indicates that weighted estimates could not be obtained because none of the lifetime cases continued to meet criteria in the twelve months prior to the interview. Subscripts indicate pairwise comparison results using multivariate logistic regressions. Groups with different subscripts are significantly different from each other. For example, African American men had lower rates of persistent Generalized Anxiety Disorder compared to both groups of Caribbean Black men (_a_ vs. _b_). However, the rates of persistent Generalized Anxiety Disorder were comparable between the U.S. and foreign born Caribbean groups (_b_ = _b_). No subscript indicates that there are no significant differences in the rate of persistence across the three groups. SE—standard error.

**Table 4:** Weighted Percent of African American vs. U.S. and Foreign Born Caribbean Black Men with History of a Lifetime DSM-IV Psychiatric Disorder That Ever Sought Mental Health Services.

DSM-IV Disorder		Lifetime DSM-IV Disorders		
		African American	Caribbean Black	
		U.S. Born	U.S. Born	Foreign Born
	N	Weighted % (SE)		
**Anxiety Disorders**				
Agoraphobia ^‡^	27	65.2 (10.9)	na	16.5 (3.1)
Post-traumatic Stress Disorder	79	74.1 (5.8) _a_	99.1 (0.4) _b_	83.2 (11.0) _a,b_
Social Phobia	115	52.8 (5.6)	72.1 (16.4)	76.5 (14.1)
General Anxiety Disorder	61	54.4 (8.0) _a_	96.8 (0.2) _b_	29.8 (17.2) _a_
Panic Disorder	47	70.6 (7.6) _a_	57.7 (37.6) _a,b_	42.1 (8.3) _b_
Panic Attack	93	51.0 (3.3) _a_	58.1 (19.3) _a_	32.9 (18.7) _b_
Any Anxiety Disorder	244	57.4 (4.0)	75.9 (12.5)	63.9 (15.6)
**Mood Disorders**				
Major Depressive Disorder	154	56.9 (5.5)	58.9 (25.8)	74.2 (10.6)
Dysthymia ^‡^	41	65.8 (6.9)	6.1 (7.2)	57.2 (17.9)
Bipolar Disorder I, II ^‡^	36	63.7 (10.7)	40.7 (19.5)	54.8 (30.4)
Any Mood Disorder	171	57.9 (5.0)	58.4 (25.3)	71.4 (11.1)
**Substance Disorders**				
Alcohol Abuse	85	52.4 (3.5) _a,b_	71.8 (16.4) _a_	63.6 (23.4) _b_
Alcohol Dependence ^‡^	81	71.1 (6.3) _a_	97.6 (1.1) _a,b_	97.4 (2.5) _b_
Drug Abuse ^‡^	157	55.2 (5.0)	55.7 (27.5)	79.9 (16.6)
Drug Dependence ^‡^	64	74.4 (6.5)	86.5 (9.7)	97.5 (3.3)
Any Substance Disorder	283	50.8 (3.5) _a,b_	71.0 (16.1) _a_	97.5 (3.3) _b_
**Any of the Above Disorders**	504	50.8 (2.7)	66.7 (14.1)	56.4 (17.1)
**2 or More of Above Disorders**	270	61.3 (3.6)	75.5 (16.0)	81.1 (10.2)

Mood Disorders include Major Depressive Disorder, Dysthymia, Bipolar Disorder I and II Anxiety Disorders include General Anxiety Disorder, Panic Disorder, Agoraphobia, Social Phobia, Post-Traumatic Stress Disorder Substance Use Disorders include Alcohol Abuse, Alcohol Dependence, Drug Abuse, Drug Dependence. Subscripts indicate pairwise comparison results using multivariate logistic regressions. Groups with different subscripts are significantly different from each other. For example, African American men with a twelve-month mood disorder have higher rates of service utilization compared to foreign born Caribbean Black men (_a_ vs. _b_). However, both U.S. born Caribbean Black men with a twelve-month mood disorder share a subscript (_a_ or _b_) with African American and foreign born Caribbean Black men, indicating they had equal rates of mental health service use. No subscript indicates that there are no significant differences in the rate of service utilization across the three groups. N—number of participants that met the criteria for a disorder during the lifetime. ^‡^ Pairwise comparisons of service use between the U.S. and foreign born Caribbean Black men were not estimated due to the small samples. na—Rates of service use could not be estimated because none of the U.S. born Caribbean Black men met the DSM-IV criteria for Agoraphobia during the lifetime. SE—standard error.
